# New Appliances & Things Medical

**Published:** 1909-01-09

**Authors:** 


### NEW APPLIANCES & THINGS MEDICAL.

### A NEW DIABETIC BISCUIT.

AKOLL BISCUITS manufactured by Messrs. Huntley and Palmer of Reading are the successful outcome of an attempt to produce a less unpalatable biscuit than usual for the use of those patients with diabetes who are upon a strict non-carbohydrate dietary. It is a matter of common knowledge among medical practitioners that most diabetic foods are exceptionally dull and unappetising. Akoll Biscuits are worthy of a trial at the hands of medical men. They are almost free from starches and sugars. On testing them we find a very small quantity of carbohydrate, probably combined with the albuminous constituents; for practical purposes this trace of reducing substance is negligible. The biscuits contain a high percentage of nitrogenous substances in a digestible form—approximately 60 per cent. of their total weight is composed of proteins; and the manufacturers state that no fewer than seven different kinds of proteins enter into their composition. Akoll Biscuits are more palatable than most diabetic foods, and are crisp and attractive in appearance. Etymologists will note that the name ἀκολλ has been chosen in order to give an indication of the special non-starchy feature of these biscuits. Like all the products of Huntley and Palmer, they are securely packed in airtight boxes with tasteful exteriors.

